# The Epigenetic Overlap between Obesity and Mood Disorders: A Systematic Review

**DOI:** 10.3390/ijms21186758

**Published:** 2020-09-15

**Authors:** Mojgan Gharipour, Majid Barekatain, Johoon Sung, Naghmeh Emami, Ladan Sadeghian, Minoo Dianatkhah, Nizal Sarrafzadegan, Shayesteh Jahanfar

**Affiliations:** 1 Isfahan Cardiovascular Research Center, Genetics and Epigenetics Department, Cardiovascular Research Institute, Isfahan University of Medical Sciences, Isfahan 8158388994, Iran; mojgharipour@yahoo.com; 2 Department of Psychiatry, School of Medicine and Behavioral Science Research Center, Isfahan University of Medical Science, Isfahan 8174673461, Iran; barekatain@yahoo.com; 3 Department Public Health Science, Genome & Health Big Data, Seoul National University, Seoul 05649, Korea; jsung@snu.ac.kr; 4 Research Department, Interventional Cardiology Research Center, Research Center, Cardiovascular Research Institute, Isfahan University of Medical Sciences, Isfahan 8158388994, Iran; naghmeh.em@gmail.com; 5 Research Department, Hypertension Research Center, Cardiovascular Research Institute, Isfahan University of Medical Sciences, Isfahan 8158388994, Iran; ladan_sadeghian@yahoo.com; 6 Research Department, Heart Failure Research Center, Isfahan Cardiovascular Research Institute, Isfahan University of Medical Sciences, Isfahan 8158388994, Iran; diantkhah.m@gmail.com; 7 Isfahan Cardiovascular Research Center, Cardiovascular Research Institute, Isfahan University of Medical Sciences, Isfahan 8158388994, Iran; nsarrafzadegan@gmail.com; 8 MPH Program, School of Public Health, Central Michigan University, Mount Pleasant, MI 48859, USA

**Keywords:** epigenetics, obesity, mood disorders, depression

## Abstract

(1) Background: Obesity and mood disorders are considered as the most prevalent morbidities in many countries. We suppose that epigenetic mechanisms may induce higher rates of obesity in subjects who suffer from mood disorders. In this systematic review, we focused on the potential roles of DNA methylation on mood disorders and obesity development. (2) Methods: This systematic review was conducted in accordance with the PRISMA statement and registered in Prospero. A systematic search was conducted in MEDLINE, Scopus, Web of Science, Cochrane Central database, EMBASE, and CINHAL. We also conducted a Grey literature search, such as Google Scholar. (3) Results: After deduplication, we identified 198 potentially related citations. Finally, ten unique studies met our inclusion criteria. We have found three overlap genes that show significant DNA methylation changes, both in obesity and depression. Pathway analysis interaction for *TAPBP*, *BDNF,* and *SORBS2* confirmed the relation of these genes in both obesity and mood disorders. (4) Conclusions: While mechanisms linking both obesity and mood disorders to epigenetic response are still unknown, we have already known chronic inflammation induces a novel epigenetic program. As the results of gene enrichment, pathways analysis showed that *TAPBP, BDNF,* and *SORBS2* linked together by inflammatory pathways. Hypermethylation in these genes might play a crucial rule in the co-occurrence of obesity and mood disorders.

## 1. Introduction

Obesity and mood disorders are considered as the most prevalent morbidities in developed and developing countries [1,2,3]. The worldwide prevalence of obesity has nearly tripled since 1975. In 2016, more than 650 million adults were obese and 38 million children under the age of 5 were overweight or obese in 2019 [4]. The prevalence of mood disorders differs based on sex and disease. For example, the prevalence of major depressive disorder (MDD) and anxiety are 17.4% and 18.2% in men, 22.7% and 23.6 in women, respectively [5].

A combination of genetics and environmental factors affect the incidence and development of obesity and mood disorders [6,7]. The type and amount of food consumed during depression appeared to be significantly correlated and could affect the weight in a long time [8]. It has been well-established that 12% of the responsible genes for obesity are shared with depression [9], and changes in the mutual pathways of the shared genes could lead to altering the pathological eating behavior in patients with mood disorders. In addition, antidepressant drugs can alter body mass indexes [10,11,12].

One of the possible biological changes that could be responsible for the co-occurrence of these disorders might be epigenetic changes [13,14]. Epigenetics could legitimize modifications in the chromatin level, which alters the expression of genes involved in obesity and mood disorder [15,16]. Epigenetics could explain complex interactions between the genome and the environment. Epigenetic modifications, such as DNA methylation and histone modification, alter DNA accessibility and chromatin structure, thereby regulating patterns of gene expression [17]. For example, increasing the methylation of DNA reduces the expression of genes, while decreasing methylation is associated with increased gene expression [18]. DNA methylation occurs in the whole genome but could play an important role in repressing gene transcription when affecting the gene promoter (especially in the CpG islands, shore, and shelves) [19]. The relationship between DNA methylation and obesity and mood disorders has been reported previously [20,21,22,23,24].

The critical question is whether epigenetic changes in overlapped genes could cause obesity and mood disorders. In other words, mood disorders, especially depression, may result in obesity through DNA methylation of the shared genes, which could affect the body composition. We hypothesized that epigenetic mechanisms might induce higher rates of obesity in subjects who suffer from mood disorders. Therefore, in this article, we focused on the potential roles of DNA methylation on mood disorders and obesity development. To answer this question, we systematically reviewed the studies investigating the methylation regions in overlap genes in patients with obesity or mood disorders. Then, we discussed possible pathways that are impressed by DNA methylation in overlap genes and possible consequent changes.

## 2. Methods

This systematic review was conducted following the PRISMA (Preferred Reporting Items for Systematic Reviews and Meta-Analyses) statement and was registered in a prospective international register of systematic reviews [PROSPERO (Prospective Register of Systematic Reviews). To find relevant articles, searches were made in MEDLINE via PubMed (www.pubmed.com; National Library of Medicine), Scopus (www.scopus.com), ISI Web of Science (www.thomsonreuters.com), Cochrane Central database, EMBASE, and CINHAL. We also searched Google Scholar (www.scholar.google.com) as Gray literature. There is no restriction regarding language, publication period, patient age (children or adult), or study design. The study identification also included manual search, based on the screening of the citations of the relevant studies.

### 2.1. Search Strategy

#### 2.1.1. Step 1: Identification of Candidate Genes for Obesity

We carried out a systematic search of DNA methylation in epigenome-wide association study (EWAS) for obesity. We reviewed EWAS study papers published until November 2019 for obesity or body mass index (BMI). All EWAS significant information such as reported genes, author(s), PubMed ID, date of publication, journal, discovery, and replication sample sizes was searched. An obesity gene was considered as a candidate gene if (1) at least one CpG site within or nearby to the gene was identified; and (2) it was functionally relevant to influence at least one of the genes related to obesity.

#### 2.1.2. Step 2: Exploration of the Role of Differentiated Methylated Obesity Genes in Mood Disorders

In the second systematic search, we conducted a literature search in the above-mentioned data-source for any epigenome-wide association with the candidate gene analysis when the study is published in the fields of mood disorders until January 2020.

#### 2.1.3. Search Term

Three groups of medical subject headings (MeSH) and non-MeSH keywords were selected to search the databases, as follows: “Obesity AND (DNA Methylation, obesity, and depression, mood disorders, bipolar, suicide”.

### 2.2. Screening

Three independent reviewers (MG, NM, MD) initially scanned titles and abstracts to select potential full-text articles for further study. When any reviewer could not exclude the title and abstract, the full text of the article was obtained via Isfahan University of Medical Sciences library. Any differences in opinion were resolved through team discussion. Inclusion or exclusion of each study was determined by discussion and consensus between the two reviews. A reference list of related articles was also checked for any missing related articles. We included cohort and case-control studies.

Data on the author(s), year of publication, sample size, study design, study cohort, experimental methods, type of tissues, candidate genes or genome, DNA purification method, DNA methylation method, DNA methylation validation, genotyping, gene expression, experimental factors, statistical methods, and significant findings were extracted independently by two reviewers. For those studies with multiple reports, a single record denoted one study with the information extracted from multiple reports. All disagreements were resolved through discussions. The reviewers endeavored to contact the original authors of the studies for any missing information in order to gather complete and consistent study information. Open-ended questions were used to prevent misleading answers.

### 2.3. Inclusion Criteria

The following inclusion criteria used: (1) Studies using EWAS approach focusing on the global DNA methylation since we aimed to find overlap methylated genes in both obesity and depression; (2) we consider all types of a mood disorders such as depression, bipolar and suicide.

### 2.4. Exclusion Criteria

Review articles, randomized clinical trials, or any paper with no quantitative data was omitted.

### 2.5. The Following Outcome Measures Were Considered

The outcome of interest was obesity, depression, psychological disorder, and suicide. We anticipated that DNA methylation levels would be reported as either categorical (DNA is either hypo-, hyper- or normally methylated) or continuous data (i.e., percentage of methylated DNA). We also searched abstracts from relevant conference papers.

### 2.6. Types of Tissue Samples Included in the Review

We decided to include methylation data regardless of the source of the sample, e.g., peripheral blood, placenta, umbilical cord blood, or buccal mucosa.

### 2.7. Format of Data Input for Factors

Risk of bias and quality assessment of selected studies were assessed through a modified Downs and Black checklist for methodological quality assessment [25]. We chose to use this checklist for quality assessment used. Additionally, this checklist provides an overall quality index as well as four sub-scales of quality assessment (reporting, external quality, internal validity-bias, and internal validity-confounding). We did not exclude any study based on quality.

## 3. Results

**Figure 1:**
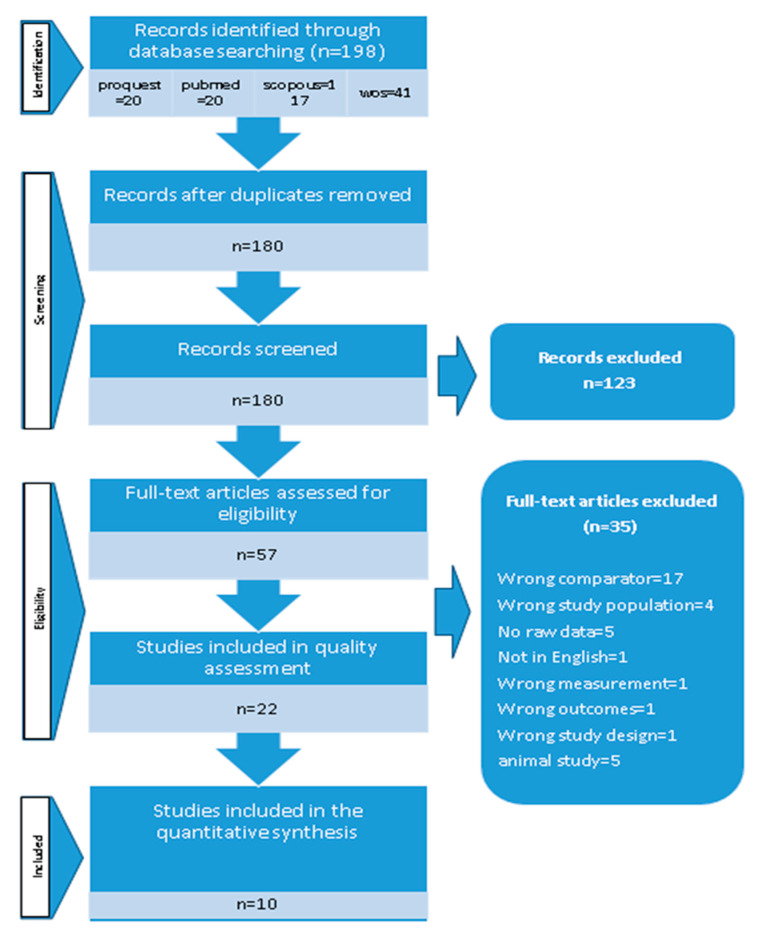
Search strategy.

After deduplication, we identified 198 potentially related citations. Based on the title and abstract, 123 studies were excluded because of inappropriate exposure (gene mutations, gene polymorphism, and microRNA), irrelevant outcomes (autoimmune diseases, cancer, and inflammation-related diseases such as asthma), or both. We also excluded investigations conducted in mice or rats. Finally, ten studies were deliberated for full-text assessment. Figure 1 shows the detailed information of the process of study selection.

**Figure 2:**
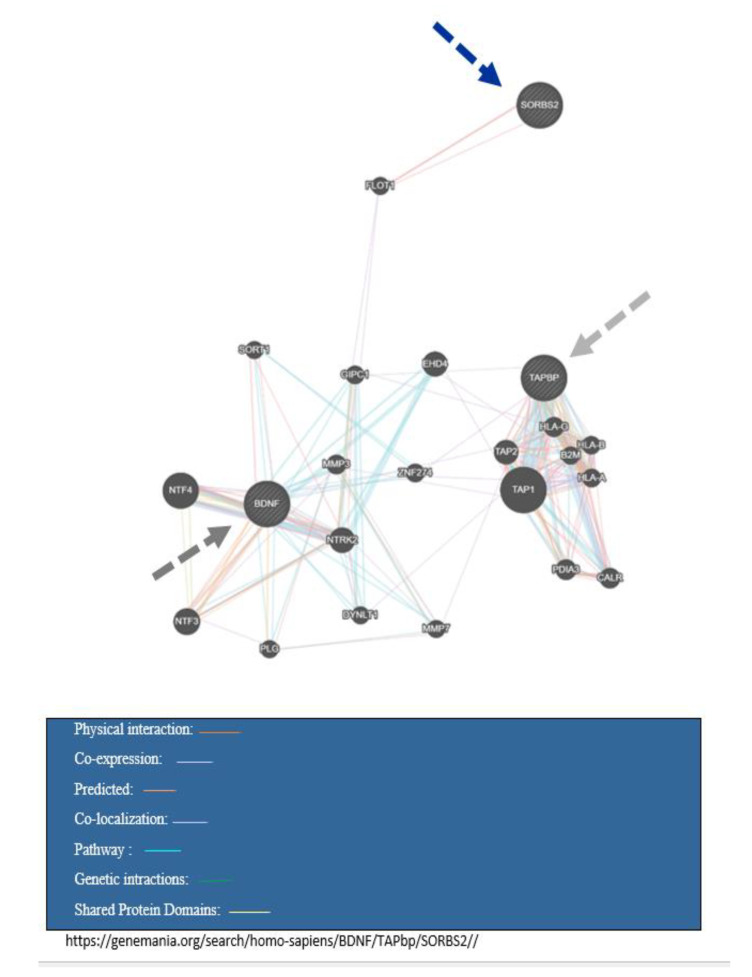
Gene interaction between overlap genes in obesity and mood disorder.

Table 1 presents a summary of the study characteristics of these selected studies. Most of the reviewed articles were published between 2014 and 2019, especially in the past four years. The selected studies mainly focused on both adults and adolescence. Most studies in this review were case-control or general population-based cohorts. There was a wide variety in terms of sample size, ranging from 5 to 115. Whole blood was the most commonly used biological sample analyzed by generally accepted DNA methylation methods, such as bisulfite conversion with pyrosequencing. Table 2 indicated the characteristics of the overlap genes. Table 3 displays a summary of biological pathways related to the *TAPBP*, *BDNF,* and *SORBS2*. Table 4 shows pathway analysis interaction for overlapped genes in obesity and mood disorders. Figure 2 demonstrate gene interaction between overlapped genes in obesity and mood disorders by the genemania software.

## 4. Discussion

To the best of our knowledge, this is the first cross-disorder systematically review that assessed the role of DNA methylation in the overlapped genes and their affected biological pathways in mood disorders and obesity. Our results revealed three overlapped genes with different methylated patterns during obesity or mood disorders, which can assist us to understand better the molecular pathophysiology of these disorders. In the further step, we attempted to identify the possible pathways that could be involved in obesity and mood disorders through the overlap genes.

In the era of the increasing prevalence of obesity and mood disorders, especially in both developing and developed world, results from our systematic review suggest an interplay between genetic susceptibility, diet, epigenetics, metagenomics, and the environment [36,37].

Evidently, obesity was found to increase the risk of depression, and depression was found to be predictive of developing obesity. Remarkably, obese persons had a 55% increased risk of developing depression over time, whereas depressed persons had a 58% increased risk of becoming obese. Neuroendocrine disturbances may also lead to depression, which in turn would cause an increase in weight over time by dysregulated stress systems or through unhealthy lifestyles. It is also possible that obesity, by its adverse effects on self-image or somatic consequences, results in the development of depression over time [38]. So, scientists struggled to find responsible genes through genome-wide association studies (GWAS) to identify the risk associated with single nucleotide polymorphisms, which might also be responsible for the co-occurrence of two conditions.

In recent years, scientific documents proved that genes are not responsible for disease by themselves, and the interaction of genes and environment is better determinants for phenotypes. Accordingly, the latest researches are likely to focus on epigenome-wide association studies (EWAS). The advantages of EWAS is considering the interaction of both genes and environments. The information gained from GWAS and EWAS has potential applications in disease control and treatment. In this study, we merely focused on DNA methylation, which could cause alterations in gene expressions and changes in the pathophysiology of diseases. We found three overlapped genes between mood disorders and obesity “*TAPBP*, *SORBS2*, and *BDNF.*” As these genes were found through published results of EWAS, we will discuss canonical pathways that might be involved in co-occurrence mood disorders and obesity.

*TAPBP:* The *TAPBP* gene is located in chromosome 6 and encodes tapsin; a transmembrane glycoprotein that mediates the interaction between newly assembled major histocompatibility complex (MHC) class I molecules. MHC1 is a transporter associated with antigen processing (TAP), which is required for the transport of antigenic peptides across the ER membrane [39,40]. *TAPBP*-mutant mice have defects in the expression of MHC class I, antigen presentation, and immune responses. Remarkably, Cui et al. found that the expression levels of HLA-ABC were upregulated even in the *TAPBP* knock-out cells by the interferon treatment, and immune rejection was reduced in *TAPBP*-deficient hESC line. Potent inflammatory molecules such as eicosanoids are able to upregulate *TAPBP* [41,42].

The important role of *TAPBP is* not recognized in the past in both obesity and mood disorders, and just in recent years. The results of EWAS-approved methylation in this gene could play a crucial role in these conditions. Murphy et al. identified epigenetic changes such as differentiated methylated regions (DMR) located in the third intron of the *TAPBP* gene that is related to the major depressive disorder and suicide [27]. Another study demonstrated hypermethylated CpG sites observed in the promoter region of *TAPBP* in obese and overweight subjects. These results confirmed by NEST cohort results revealed differentially methylated CpGs of *TAPBP* gene is related to the maternal pre-pregnancy obesity [28]. In vitro experiments revealed higher methylation levels of *TAPBP,* such as those found in above-mentioned studies might decrease tapsin via reduced transcriptional activity, leading to impaired immune responses and lower CD8 + T-cell responses [43,44,45]. In mice, tapsin is activated by the cytokines like IFN-γ and IFN-β, and to a lesser extent, TNF-α [45].

These results were very thought-provoking and cited several times by others and unlocked doors to the diagnosis of pathophysiology and new treatments.

*TAPBP* is linked to both mood disorders and obesity through the JNK pathway. This pathway plays a vital role in the inflammatory response and oxidative stress [43]. Briefly, stress-induced JNK activation occurs in the adipose and liver tissue of obese mice, whether obesity is induced by a high-fat diet or genetically through leptin deficiency (obese/obese mice). Insulin resistance in obese mice through ER stress-mediated JNK pathway is induced by the phosphorylation of insulin receptor substrate 1 (IRS1), which impairs insulin action and causes insulin resistance [44].

Interestingly, in the different tissues of obese subjects, inflammatory factors can be observed to cause continuous activation of JNK. The activated JNK acts on nuclear factor-κB (NF-κB) and activator protein-1 (AP-1) to produce more inflammatory factors, further reducing the sensitivity of insulin target cells toward insulin, finally forming a vicious circle and aggravating insulin resistance. Moreover, a network framed by PPARγ, NF-κB, and PTP1B signaling pathways crossing with the JNK signaling pathway plays a crucial role in regulating insulin resistance [39].

We assumed that a better understanding of the JNK signaling pathway and its relationship with PPARγ, NF-κB, PTP1B signaling pathways are necessary for a new drug targeting the treatment of obesity and mood disorders [39].

*SORBS2*: The role of *SORBS2* gene in obesity and mood disorders has been discovered recently by different genome-wide methylation studies [30]. This gene located on the 4q35. 1 encodes the Arg protein tyrosine kinase binding protein 2 (ArgBP2). *SORBS2* is an RNA-binding protein, which is involved in the regulation of RNA metabolism [46]. *SORBS2* is involved in several biological pathways (Table 2). Sorbin, the product of *SORBS2,* is an ArgBP2 protein and SH3 domain-containing protein 2 and might be involved in insulin-mediated translocation of GLUT4 and thereby might affect energy storage [47]. Previous research has highlighted the role of this functional protein in disease states [48,49,50,51]. Downregulation of this gene was reported to be associated with mood disorders [52]. Linear regression analyses revealed a strong association of methylation with BMI for *SORBS2* in abdominal omental visceral adipose tissue [53]. There is enough data to provide functional evidence that promoter methylation in *SORBS2* directly influences gene activity and thus contributes to the abiogenesis. We suggest that *SORBS2* is related to obesity through the innate immunity and inflammation response by the Notch signaling pathway that plays a major role in adipogenic differentiation [54]. Increased Notch signaling in mice blocked the expansion of white adipose tissue, ectopic fat accumulation, and insulin resistance [55].

The genetic deletion of Sorbin in mice leads to mood disorders by a reduction in the average number of spines per dendrite [49]. Additionally, to the grapevine, *SORBS2* is related to mood disorders through two different pathways; actin-related proteins and the Notch signaling pathway [56,57]. Notch signaling is important in regulating neural cell proliferation, differentiation, and neural cellular growth, and is considered as a contributor in adaptive and innate immune responses. Active Notch signaling has been observed under a variety of inflammatory conditions such as atherosclerosis [55,58]. Interestingly, prototypical proinflammatory cytokines positively regulate Notch signaling and its target gene expression. For example, TNF induces expression of Notch1, Notch4 [59]. In addition, IL-1β induces Notch target genes, and Interferon-γ (IFNγ) functions as a negative regulator of Notch pathway activation [60].

*BDNF:* This gene is located in the 11p14.1 and encodes a member of the nerve growth factor family of proteins [61]. Alternative splicing results in multiple transcripts, at least one of which encodes a preproprotein that is proteolytically processed to generate the mature protein. Binding of this protein to its cognate receptor promotes neuronal survival in the adult brain. *BDNF* gene structure is complex, regulated by nine functional promoters. Each promoter regulates the expression of this gene [62]. *BDNF* encompasses several biological pathways (Table 2) and has a complex regulation; the exact roles of *BDNF* and its transcripts are not fully understood. BDNF insufficiency or missense mutations in its receptor, TrkB, are associated with weight gain and obesity in humans and mouse models [63,64]. In line with these observations, both exogenous *BDNF* administration and *BDNF* gene transfer in mouse model support the concept of the BDNF deficit in the brain induces a metabotropic impairment leading to obesity. Essentially, it has been established that the hypothalamic reduction of *BDNF* modulates energy homeostasis affecting food intake and promoting an anorectic signal [65].

There are several pieces of evidence about the role of BDNF in brain function and mood disorders [66,67,68]. Previous studies indicated that the positive correlation between brain and circulating BDNF suggests that BDNF levels in the blood reflect the levels occurring in the central nervous system. Thus, circulating BDNF has been proposed as a potential biomarker for neuropsychiatric disorders and neurodegenerative diseases [69,70,71,72,73,74].

BDNF is one of the major neurotrophic factors, plays an important role in the maintenance and survival of neurons and in synaptic plasticity. Several lines of evidence suggest that BDNF is involved in depression and plays an important role in the maintenance and survival of neurons and in synaptic plasticity. Recent documents demonstrated that the expression of *BDNF* is decreased in depressed patients [75]. BDNF has a multifaceted role from its neurotrophic activity to inflammation, metabolism, and cardiovascular diseases [76,77,78].

Methylation of the *BDNF* gene was analyzed at CpG sites in upstream of exon I. It is also possible that the hypomethylation promotor is located in exon I, which could cause altered *BDNF* expression, leading to abnormal eating behaviors [35,79]. Gardner et al. displayed different methylation in the promoter of *BDNF* related to obesity [35]. Interestingly, three of the obesity-associated CpGs were located within two of the numerous promoters of *BDNF*, and differential *BDNF* transcripts are expressed at different time points and in different cellular compartments [80,81]. Carriers of the risk allele at rs10767664 had higher methylation in the pII promoter of *BDNF*, and lower methylation in the pVI promoter of *BDNF* [31]. Januar et al. have revealed that late-life depression is associated with elevated *BDNF* methylation of specific CpG sites within promoters I and IV, with all associations remaining after adjustment for a range of covariates [33].

Furthermore, recent studies reported an increased *BDNF* methylation is associated with depression in animal models [82] and in humans [83]. Decreased BDNF may relate to the reduced function of the *BDNF* gene in promoting neural growth and repair in depression. Thus, among depressive patients, those with a higher *BDNF* methylation status are at a greater risk of suicidal behavior [84]. Hypermethylation in Exon I, in the promoter region, reduced BDNF levels in the plasma and post-mortem hippocampus of depressed individuals [85,86,87,88]. Another post-stroke cohort indicated that higher *BDNF* promoter methylation status was independently associated with depressive symptoms over one year after the onset of stroke, although not associated with baseline depressive symptom severity [84,89,90]. The methylation state of CpG sites within mouse promoter/exon IV is correlated with the expression of BDNF in the developing mouse forebrain, and similar associations were found with chronic depression, and these effects were not driven by antidepressant treatment [69]. For example, Jin et al. using the Sequenom Mass Array platform, demonstrated in mice model that fluoxetine can downregulate the expression of BDNF by the methylation of 11 CpG sites in promoter IV [91].

Strangely, BDNF has leading biological roles in inflammation and apoptosis; consequently, it is a crucial neurotrophic factor for preserving normal nervous system function. Moreover, BDNF is an associated member of the neurotrophic factor family that is mainly secreted by neuron or glial cells [92].

Sources of chronic inflammation or non-resolving inflammation may originate from either pathophysiological (e.g., inflammatory diseases, immune-based disorders, T cell dysfunction) or non-pathological conditions, including aging and obesity. Interestingly, BDNF has main biological roles in inflammation and apoptosis; thus, it is a crucial neurotrophic factor for preserving normal nervous system function [92].

Additionally, BDNF has a multifaceted role from its neurotrophic activity to inflammation, metabolism, and cardiovascular diseases. BDNF is considered as a potential modulator/mediator with anti-inflammatory effects [86].

BDNF-related neuroprotective effects are elicited by activation of extracellular signal-related kinase (ERK) and phosphatidylinositol 3-kinase (PI3K)/protein kinase B (AKT)-signaling pathways. Production of inflammatory cytokines can regulate by complex signaling pathways, especially nuclear factor-κb (NF-κB) and inflammatory response signal pathway (BDNF-TrkB-MEK-ERK-NF-κB pathway) [93,94,95].

## 5. Limitation

This study strengthens the novel findings related to the overlap genes in obesity and mood disorders but is limited in accessing row epigenome data to do gene enrichment analysis. None of the authors of the included studies were interested in responding to our inquiry to share their raw data to do a meta-analysis.

## 6. Conclusions

While mechanisms linking both obesity and mood disorders to epigenetic response are still unknown, it is well-known that chronic inflammation induces a novel epigenetic program. As the results of gene enrichment pathways analysis exhibited that *TAPBP, BDNF,* and *SRBP2* are related together by inflammatory pathways, we hypothesis that hypermethylation in these genes might play a crucial role in the co-occurrence of obesity and mood disorders due to the inflammation process. Our results shed light on our understanding of such associations. Future studies should focus on the molecular pathophysiology of these disorders in the hope of opening new approaches for target treatment.

## Figures and Tables

**Table 1:** Genomic regions investigated in reviewed studies.

Authors	Gene	Outcome	Tissue Type	Study	Population	Groups	Definition	Method
Cheng (2018)	TAPBP	Obesity	WBC	Case-Control	62 years old and older	Obese/overweight: (*n* = 5) Normal weight: (*n* = 5)	BMI < 25 kg m^−2^, overweight, 25 ≤ BMI < 30 kg m^−2^, and obese, BMI ≥ 30 kg m^−2^	Epigenome-wide DNA methylation was analyzed using the Infinium Human Methylation450 (HM450) BeadChip (Illumina, San Diego, CA, USA)
Murphy (2017)	TAPBP	Depression	Tissue brain	Case-Control	Adults	Tissue (*n* = 75) from two regions of the cortex (BA11, *n* = 40 BA25, *n* = 35)	Psychological autopsy method	Differential DNA methylation across the PSORS1C3-associated DMR (spanning a region)
Martin (2019)	TAPBP	Obesity	Cord blood	Case-Control	18 years and older	Pre-pregnancy obesity in 187 mother-female and 173 mother-male offsprings	Infant birth weight and sex.	differential methylation at 6148 CpG sites (FDR) using Illumina umanMethylation450k BeadChip
Rhee (2017)	SORBS2	Obesity	WBC	Case-Control	Twins Children	Obese children = 6 Normal body weight = 6	Normal weight, BMI < 25 kg m^−2^, Obese and overweight (*n* = 5, BMI ≥ 25 kg m^−2^) versus Normal weight group (*n* = 5, BMI < 25 kg m^−2^)	Illumina Human HT-12 v4 Expression BeadChip Kit, with 47,318 probes.
Zhu (2019)	SORBS2	Depression	WBC	Case-Control	Twins 18 and older	79 monozygotic twin pairs discordant	MDD diagnoses were determined using the Structured Clinical Interview for DSM-IV Research Version (SCID-4-RV)	Integrative DNA methylome and transcriptome analysis Infinium HumanMethylationEPIC BeadChip (Illumina Inc., CA, USA)
Keller (2010)	SORBS2	Obesity	Adipose tissue	Case-Control	18 and older	Men (N¼39) and women (N¼66)		Differential methylation analysis using InfiniumHumanMethylation450 BeadChips
Perroud (2013)	BDNF	Bipolar	WBC	Case-Control	18 years and older	Control = 52 Bipolar disorder = 115	1.Suicidal or para-suicidal behaviors 2. Severe impulse control disorders 3. Anger problems 4. Receiving psychopharmacological treatment 5. Fulfilling DSM-IV (diagnostic and statistical manual of mental disorders, 4th edition)	Selected region/gene/high resolution melting method
Januar (2015)	BDNF	Depression	Buccal tissue	Case-Control	65 years	Depressive = 251 Non-depressive = 773	Diagnostic and Statistical Manual of Mental Disorders-IV criteria and using the Mini International Neuropsychiatric Interview (MIN)	Sequenom Mass ARRAY (San Diego, CA, USA)
Voisin (2015)	BDNF	Obesity	WBC	Case-Control	14–16 years	Two sub-groups of healthy young Caucasians from two different age ranges	Lean: BMI < 25 Overweight: 25 ≤ BMI < 30 Obese: BMI ≥ 30	Genome-wide Illumina Infinium human Methylation450 Bead Chip (Illumina)
Gardner (2015)	BDNF	Obesity	WBC	Case-Control	Children	32 non-obese and 32 obese African-American children aged 5–6 years.	Normal weight (BMI-for-age percentile 5th–<85th) or obese (BMI for-age percentile ≥ 95th).	Methylation-sensitive restriction enzyme digestion 2.qRTPCR

WBC: White blood cells.

**Table 2:** A summary of biological pathways related to the *TAPBP, BDNF*, and *SORBS*.

	Biological Process (GO)	Molecular Function (GO)	Cellular Component (GO)	KEGG Pathways	Super Pathway	Ref.
**TAPBP**	Antigen processing and presentation of peptide antigen via MHC class I Antigen processing and presentation of exogenous peptide antigen via MHC class I, TAP-dependent Antigen processing and presentation of exogenous peptide antigen Antigen processing and presentation of endogenous peptide antigen Antigen processing and presentation of endogenous peptide antigen via MHC class I	TAP binding Peptide Antigen Binding Peptide Binding MHC protein binding Peptide Antigen-Transporting ATPase Activity	Phagocytic Vesicle Membrane Integral Component of Lumenal Side of Endoplasmic Reticulum Membrane Integral Component of Endoplasmic Reticulum Membrane MHC Class I Protein Complex	Antigen processing and presentation Herpes simplex infection HTLV-I infection Graft-versus-host disease	Antigen Processing-Cross presentation ER-Phagosome pathway Immune response Antigen presentation by MHC class I Human cytomegalovirus infection Human immunodeficiency virus 1 infection Class I MHC mediated antigen processing and presentation	https://string-db.org/cgi/network.pl?taskId=BgKUv6snBF5M
**SORBS2**	Developmental cell growt Molecular Function (GO) Protein kinase binding Mitogen-activated protein kinase binding Ephrin receptor binding Phosphotyrosine residue binding receptor tyrosine kinase binding	Protein Kinase Binding Mitogen-Activated Protein Kinase Binding Ephrin Receptor Binding Phosphotyrosine Residue Binding R Binding	Contractile Fiber Part Myofibril Actin Cytoskeleton Focal Adhesion Podosome	Chronic myeloid leukemia Bacterial invasion of epithelial cells ErbB signaling pathway Shigellosis Neurotrophin signaling pathway	Notch signaling pathway Actin filament organization Biological process Cell growth involved in cardiac muscle cell development	https://string-db.org/cgi/network.pl?taskId=kpCKo7eP0PWT
**BDNF**	Neurotrophin TRK receptor signaling pathway Transmembrane receptor protein tyrosine kinase signaling pathway Regulation of neuron death Regulation of cell deathNegative regulation of neuron death	Neurotrophin Binding Neurotrophin Receptor Binding Receptor Binding Activity Nerve Growth Factor Binding Cellular Component (Go)	Neuron Projection Cytoplasmic Vesicle Postsynaptic Membrane Dendrite Axon	Neurotrophin signaling pathway MAPK signaling pathway PI3K-Akt signaling pathway Cocaine addiction	Cellular apoptosis pathway mitochondrial apoptosis Apoptotic Pathways in Synovial Fibroblasts p53 Mediated Apoptosis DHA Signaling Telomerase Components in Cell Signaling PPAR Pathway Rac1 Pathway Glioma Invasiveness Actin-Based Motility by Rho Family GTPases ERK5 Signaling eIF2 Pathway Rap1 Pathway Nuclear Receptor Activation by Vitamin-A Paxillin Interactions Ras Pathway GPCR Pathway Pancreatic Adenocarcinoma Breast Cancer Regulation by Stathmin1 NFAT in Immune Response Estrogen Pathway ERK Signaling Rho Family GTPases MAPK Signaling Molecular Mechanisms of Cancer ILK Signaling GSK3 Signaling Nanog in Mammalian ESC Pluripotency 3-3-14 Induced Intracellular Signaling eNOS Signaling CREB Pathway IP3 Pathway Activation of PKC through GPCR Intracellular Calcium Signaling BDNF-TrkB Signaling ERK Pathway in Huntingtons Disease Follicle Stimulating Hormone (FSH) signaling pathway	https://string-db.org/cgi/network.pl?taskId=MU4AHU3o8Jwe

**Table 3:** Pathway analysis interaction for overlap genes in obesity and mood disorder.

Genes	Term	*p*-Value	Adjusted *p*-Value	Odds Ratio	Combined Score
**TAPBP**	Antigen Presentation: Folding, assembly and peptide loading of class I MHC_Homo sapiens_R-HSA-983170	0.00125	1	800	5347.706
	ER-Phagosome pathway_Homo sapiens_R-HSA-1236974	0.00325	1	307.6923	1762.805
	Antigen processing-Cross presentation_Homo sapiens_R-HSA-1236975	0.0041	1	243.9024	1340.679
	Class I MHC mediated antigen processing & presentation_Homo sapiens_R-HSA-983169	0.01525	1	65.57377	274.3072
	Adaptive Immune System_Homo sapiens_R-HSA-1280218	0.0381	1	26.24672	85.76236
	Immune System_Homo sapiens_R-HSA-168256	0.07735	1	12.92825	33.08879
**SORBS2**	Extracellular vesicles in the crosstalk of cardiac cells WP4300	9.50 × 10^−4^	0.44839	1052.632	7325.339
**BDNF**	ERK Pathway in Huntington’s Disease WP3853	7.00 × 10^−4^	0.330392	1428.571	10377.79
	Follicle Stimulating Hormone (FSH) signaling pathway WP2035	0.00135	0.318593	740.7407	4894.571
	BDNF-TrkB Signaling WP3676	0.0017	0.267461	588.2353	3751.263
	Synaptic signaling pathways associated with autism spectrum disorder WP4539	0.0025	0.294995	400	2396.593
	Prader-Willi and Angelman Syndrome WP3998	0.00305	0.287915	327.8689	1899.223
	MECP2 and Associated Rett Syndrome WP3584	0.0031	0.243863	322.5806	1863.345
	Spinal Cord Injury WP2431	0.0059	0.397824	169.4915	869.9687
	Brain-Derived Neurotrophic Factor (BDNF) signaling pathway WP2380	0.0072	0.424795	138.8889	685.2341
	Sudden Infant Death Syndrome (SIDS) Susceptibility Pathways WP706	0.0079	0.414306	126.5823	612.7726
	MAPK Signaling Pathway WP382	0.0123	0.580555	81.30081	357.5744
	PI3K-Akt Signaling Pathway WP4172	0.017	0.729449	58.82353	239.6794

**Table 4:** Report of Black and down score.

Authors	Reporting Score	External Validity	Internal Validity-Bias	Internal Validity-Confounding	Black and Dwaon Score
Cheng, et al. (2018) [26]	6	2	1	2	11
Murphy, et al. (2017) [27]	5	1	0	0	6
Martin, et al. (2019) [28]	7	2	1	3	13
Rhee, et al. (2017) [29]	7	2	0	0	9
Zhu, et al. (2019) [30]	6	2	1	2	11
Keller, et al. (2010) [31]	4	0	1	0	4
Perroud, et al. (2013) [32]	7	1	1	0	9
Januar, et al. (2015) [33]	4	2	1	1	8
Voisin, et al. (2015) [34]	7	2	0	2	11
Gardner, et al. (2015) [35]	6	1	1	2	10

## Abbreviations

ArgBP2	Arg/c-Abl kinase binding protein 2
BMI	body mass index
DMR	differentiated methylated regions
ER	endoplasmic reticulum
EWAS	Epigenome wide association study
IRS1	insulin receptor substrate 1
MDD	Major Depressive Disorder
MHC	major histocompatibility complex
OVAT	omental visceral adipose tissue
PRISMA	Preferred Reporting Items for Systematic Reviews and Meta-Analyses
PROSPERO	Prospective Register of Systematic Reviews
SOHOs	orbin homology
TAP	transporter associated with antigen processing
